# Revealing third-order interactions through the integration of machine learning and entropy methods in genomic studies

**DOI:** 10.1186/s13040-024-00355-3

**Published:** 2024-01-30

**Authors:** Burcu Yaldız, Onur Erdoğan, Sevda Rafatov, Cem Iyigün, Yeşim Aydın Son

**Affiliations:** 1 Department of Health Informatics, Graduate School of Informatics, METU, Ankara, Turkey; 2 Department of Industrial Engineering, METU, Ankara, Turkey; 3 Graduate School of Informatics, ODTU-NOROM, METU, Ankara, Turkey

**Keywords:** Biomarker, Three-way interaction, Entropy, GWAS, Alzheimer disease

## Abstract

**Background:**

Non-linear relationships at the genotype level are essential in understanding the genetic interactions of complex disease traits. Genome-wide association Studies (GWAS) have revealed statistical association of the SNPs in many complex diseases. As GWAS results could not thoroughly reveal the genetic background of these disorders, Genome-Wide Interaction Studies have started to gain importance. In recent years, various statistical approaches, such as entropy-based methods, have been suggested for revealing these non-additive interactions between variants. This study presents a novel prioritization workflow integrating two-step Random Forest (RF) modeling and entropy analysis after PLINK filtering. PLINK-RF-RF workflow is followed by an entropy-based 3-way interaction information (3WII) method to capture the hidden patterns resulting from non-linear relationships between genotypes in Late-Onset Alzheimer Disease to discover early and differential diagnosis markers.

**Results:**

Three models from different datasets are developed by integrating PLINK-RF-RF analysis and entropy-based three-way interaction information (3WII) calculation method, which enables the detection of the third-order interactions, which are not primarily considered in epistatic interaction studies. A reduced SNP set is selected for all three datasets by 3WII analysis by PLINK filtering and prioritization of SNP with RF-RF modeling, promising as a model minimization approach. Among SNPs revealed by 3WII, 4 SNPs out of 19 from GenADA, 1 SNP out of 27 from ADNI, and 4 SNPs out of 106 from NCRAD are mapped to genes directly associated with Alzheimer Disease. Additionally, several SNPs are associated with other neurological disorders. Also, the genes the variants mapped to in all datasets are significantly enriched in calcium ion binding, extracellular matrix, external encapsulating structure, and RUNX1 regulates estrogen receptor-mediated transcription pathways. Therefore, these functional pathways are proposed for further examination for a possible LOAD association. Besides, all 3WII variants are proposed as candidate biomarkers for the genotyping-based LOAD diagnosis.

**Conclusion:**

The entropy approach performed in this study reveals the complex genetic interactions that significantly contribute to LOAD risk. We benefited from the entropy-based 3WII as a model minimization step and determined the significant 3-way interactions between the prioritized SNPs by PLINK-RF-RF. This framework is a promising approach for disease association studies, which can also be modified by integrating other machine learning and entropy-based interaction methods.

**Supplementary Information:**

The online version contains supplementary material available at 10.1186/s13040-024-00355-3.

## Background

Genome-wide association Studies (GWAS) explore the statistical association of the SNPs in complex genetic disorders using high-dimensional datasets [1]. Primarily, these associations are identified with single-locus approaches, whereby each SNP is tested individually for the association. However, the univariate approach could not explain a large proportion of genetic heritability in most complex diseases. Interactions at higher dimensions, such as SNP-SNP, gene–gene, and gene-environment interactions, can address the missing heritability problem [2].

While some of these interactions are identified in small-scale studies, most are revealed in Genome-Wide Interaction Studies (GWIS). A variety of machine learning methods, such as multifactor dimensionality reduction (MDR) and random forest (RF), are used to disclose the complex interactions of the variants [3–6]. Also, Entropy-based methods have been proposed to analyze non-linear relationships between genotypes in complex diseases [7]. Various entropy-based approaches for pairwise, third-order, and high-order interactions have been suggested for different study designs, such as family-based, case-only, and case–control [8–12].

However, detecting 3-way and K-way interactions by exhaustive examination requires considerable computational resources. Therefore, Cantor et al. have recommended prioritizing SNPs most associated with a trait before assessing the interactions [13]. In this context, entropy-based methods demonstrate the statistical interactions for the SNPs selected by different machine learning models [13, 14].

Alzheimer Disease (AD) is a progressive neurodegenerative disorder that is the most common cause of late-onset dementia. More than 55 million people live with dementia worldwide currently, and it is estimated that AD may contribute to 60–70% of the cases [15]. AD is characterized by cognitive impairment, but significant heterogeneity can be observed in clinical progression. It is depicted as early-onset (EOAD) and late-onset (LOAD) based on the age of onset, and LOAD constitutes approximately 95% of cases. While EOAD is known to be familial and inherited in a Mendelian pattern, LOAD presents complex genetic inheritance, where interactions of multiple genetic variations and environmental factors affect the phenotype of patients [16]. In early studies, APOE4 was established as a genetic risk factor for LOAD [17–19]. Several GWA studies have revealed several risk variants in recent years [20–25]. Besides, various studies have identified epistatic interactions [14, 26–31]. However, only two-way interactions have been considered in previous studies.

This study aims to detect the variants with third-order interactions in LOAD by calculating the total information common to all three attributes but not present in any subset (3WII) in a case–control study design. We integrated a multi-step machine learning approach and an entropy-based 3-way interaction information method proposed by Fan et al. An extensive set of SNPs prioritized by PLINK-RF-RF analysis of the LOAD GWAS datasets are analyzed without mapping them to individual genes to reduce the bias. Then, the significant SNP combinations are identified by using entropy-based test statistics. These prioritized SNP combinations are proposed as potential early and differential diagnosis markers.

## Methods

### Data

Three different high-dimensional datasets from the Alzheimer Disease Neuroimaging Initiative (ADNI), GenADA, and the National Centralized Repository for Alzheimer Disease and Related Dementias (NCRAD) are obtained via dbGaP control access [32, 33]. Affymetrix Mapping250K_Nsp and Mapping250K_Sty Illumina Human610_Quadv1_B 500K and Illumina Human610-Quad BeadChip platforms are used by these initiatives, and 620,901, 410,907, and 585,295 QC passed SNPs were included in this study respectively. 210 controls and 344 cases for ADNI, 777 controls and 798 cases for GenADA, and 1310 controls and 1289 cases for NCRAD are genotyped using these platforms.

### PLINK

Initial analysis was done for the dimension reduction to eliminate the statistically non-significant SNPs before building a LOAD model from each dataset. PLINK analysis was run using the “–assoc” function for identifying the independent statistical significance of variations in association with the LOAD [34, 35]. SNPs were filtered by the 0.01 basic allelic test chi-square *p*-value threshold. In general, we did not account for potential covariates like gender, age, and population structure because we utilized PLINK results primarily for filtering and eliminating non-disease-related SNPs, which served as our initial dimension reduction step in the workflow. The *p*-value threshold was not utilized according to traditional statistical power-providing methods, as this would be too stringent. Instead, we opted for a more adaptable *p*-value to supply the machine-learning model with an appropriate dataset size adequately.

### SNP selection with RF-RF approach

After filtering with PLINK, SNPs significantly associated with LOAD were used as input for the multi-step RF modeling. RF is a supervised learning method with collections of decision trees that build a better predictive performance than a single classification and regression model [36]. One of the critical elements in understanding the role of individual features (SNPs) in the RF model is feature importance. Feature importance in RF is calculated based on the permutation importance that denotes the random permutation worked better than the original. It can be inferred that the variable does not have a role in the prediction is unimportant. This measure quantifies the extent to which a feature contributes to the overall predictive performance of the model. In other words, the features that improve the purity lead to more considerable information gain. To enhance the interpretability of our model, we should clarify that higher feature importance values indicate that a particular SNP plays a more critical role in the decision-making process of the RF model. We can identify SNPs significantly impacting the model’s predictions by considering feature importance by permutated value. We used the initial RF for dimension reduction by using feature importance via permutation test and the second RF step to model and validate the selected attributes from the initial RF. RF algorithm was implemented by using a 5-fold cross-validation (CV) technique to reduce overfitting. In addition, the most important part, which is called prioritization of significant attributes related to AD, the RANGER package in R [37], was used. As reported in the results section, model tuning was performed on “mtry” and “ntree” parameters in the modeling and validating phase. For an increase in performance with ntree, the larger value of mtry is selected. The ‘mtry’ parameter is a pivotal component in addressing correlated features. It controls the number of features considered for splitting at each node of the decision trees within the ensemble. It essentially dictates the diversity of feature selection, which is particularly relevant in the context of correlated features. Accordingly, “mtry” values were selected to create a manual grid. This grid was created with the SNP count’s square root and the square root’s fold. RF tuning split rule was selected to be the “gini index”. Each decision tree in the forest was created as a tree of maximum size. Importance and importance *p*-values were also calculated, and after the first RF, features with an importance value smaller than 0.05 were selected for the following modeling step. This multi model approach was implemented for each dataset for prioritization.

### Entropy-based prioritization

Fan. et al. proposed an information gain approach based on mutual information for two-way interactions and used an interaction-information gain approach for three-way and higher-order interactions [10]. They also develop one-dimensional test statistics to analyze sparse data for investigating 2-way, 3-way, and K-way interactions in case–control settings. The prioritized SNPs in each dataset are investigated in three-way interactions, as described below.

Two-way mutual information and three-way interaction information are entropy-based methods that measure the interaction between two markers and the information common to all three attributes. D = 0 denotes the disease status of an individual for healthy individuals, and D = 1 for affected ones in a case–control study design. The difference between the mutual information in the affected population and the general population is defined as information gain:

$${\text{IG}}({\text{X}},{\text{Y}}\backslash {\text{D}})={\text{I}}({\text{X}},{\text{Y}}\backslash {\text{D}})-{\text{I}}({\text{X}},{\text{Y}})$$

Interaction information gain of markers X, Y, and Z are defined similarly:

$${\text{IIG}}({\text{X}},{\text{Y}},{\text{Z}}\backslash {\text{D}})={\text{I}}({\text{X}},{\text{Y}},{\text{Z}}\backslash {\text{D}})-{\text{I}}({\text{X}},{\text{Y}},{\text{Z}})$$

Information gain-based test statistic (T_IG_) is calculated by dividing IG or IIG by a specific normalization factor of variance Ʌ. The resulting test statistics are centrally chi-square distributed with 1 degree of freedom under the null hypothesis that the markers are independent of the disease.

The SNPs prioritized by the LOAD-RF-RF model were filtered from BED files and divided into case–control groups using PLINK functions. Then, the genotype frequencies of SNP pairs and triplets were calculated using a custom Python script [38]. These frequencies were used as parameters for 3-way interaction information gain and two-way mutual information gain functions for identifying SNPs that would explain the susceptibility of LOAD. These two functions were implemented using custom R scripts adapted from Fan R. [39]. We calculated the test statistic based on the interaction information gain (IIG) for prioritized SNPs in each dataset. Significantly different interactions were identified by using *p*-values assigned in these test statistics. Then, two-way mutual information gain test statistics were calculated for the triplets’ variants, which were found to have significant interactions in the previous step. Since we looked for the interactions common to all three variants that cannot be explained by two-way mutual information gain, the triplets with SNP combinations with significant two-way mutual information gain were excluded.

### Multiple test correction

Permutation testing validated significant interactions for multiple comparisons [40]. Disease status labels were randomly shuffled, and information gain-based test statistic was calculated in each iteration for performing 1000-fold permutation testing for GenADA and ADNI datasets. 10000-fold permutation testing was performed for the NCRAD dataset to accommodate prioritized SNPs and triplets, which were more significant than the other datasets. Then, the ratio of test statistics greater than the observed test statistics was calculated to assign a *p*-value to the permutation testing.

Overall, the entropy analysis of the PLINK-filtered, RF-RF prioritized variant set reveals the 3-way interactions. After the permutation testing, the significant triplets are filtered based on the permutation *p*-value. The triplets with a *p*-value greater than 0.05 were filtered out in the GenADA and the ADNI datasets. Triplets with permutation *p*-value > 0.01 are filtered out in the NCRAD dataset.

### Variant annotation

**Fig. 1:**
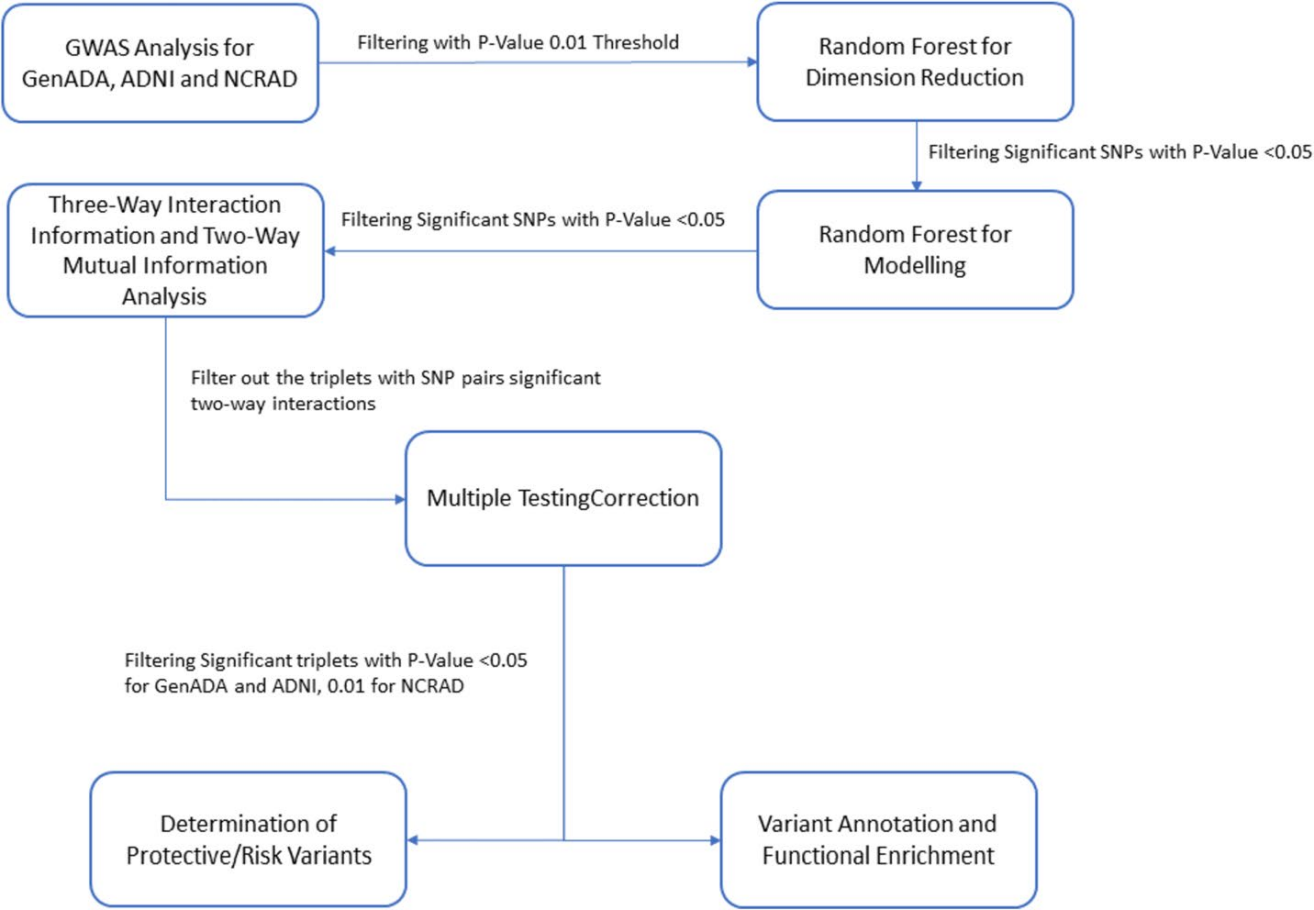
Overview of the methodology

SNPNexus and SNiPA tools have been used to annotate the variants in the filtered triplets [41–45]. Genomic mapping, variant annotation, gene/protein consequences, and phenotype/disease association information have been obtained from these tools (Fig. 1).

### Functional enrichment

GO Molecular Function, GO Cellular Component, GO Biological Process, and Reactome pathways are analyzed using the g: GOSt component of the g: Profiler tool [46] for the variants reported in each triplet for all datasets. All analyses have been done with default attributions with a significance threshold 0.05. The *p*-value of the enrichment of pathways has been computed using Fisher’s exact test, and the Bonferroni correction method has been used for multiple testing corrections.

Then, EnrichmentMap [47], a plugin for the Cytoscape tool [48], has been used to create networks from Gene Ontology annotations and Reactome pathways. All analyses have been done with a *p*-value of 0.05, FDR q-value cutoff of 0.01, and edge similarity cutoff of 0.3 (Jaccard metric).

## Results

### Feature selection by LOAD-RF-RF model

After PLINK association analysis is performed for each controlled accessed GWAS dataset, significance values are calculated to compare allele frequencies between cases and controls. A total of 7639 SNPs for ADNI, 3767 SNPs for GenADA, and 16404 SNPs for NCRAD with *p*-values smaller than 0.01 were selected. We found only one common SNP for these datasets. 18 common SNPs between NCRAD and GenADA, 206 common SNPs between NCRAD and ADNI, and nine common SNPs between ADNI and GenADA were observed.

The RF model is implemented for the feature selection among filtered SNPs based on the PLINK association for each dataset. For GenADA, the model is tuned with mtry values 2, 5, 10, 20, 41, 83, and 166. Considering the diagnostic model error rate with the 5-fold cross-validation methodology, the best mtry and ntree parameters are determined as 2 and 900, respectively (Supplementary Figure 1). The mtry parameter values 2, 4, 9, 19, 39, 78, 157, and ntree parameter values up to 1000 are used for model tuning in the ADNI dataset, and mtry = 39, ntree = 50 is determined as the best model parameters (Supplementary Figure 2). The mtry values 5, 10, 20, 41, 83, 166, and 333 are used for NCRAD dataset RF model tuning. The mtry and ntree parameters were determined as 83 and 1000 as the best model parameters (Supplementary Figure 3). The permutation hypothesis test calculates the contribution of random change in the value of the variation to the accuracy rate. After 100 permutations, 390 variants from ADNI, 1740 from NCRAD, and 434 from GenADA datasets related to the disease were selected as the input set for the modeling step with second RF at a 95% confidence level (Type I error = 0.05).

In the last step of the multi-step LOAD-RF-RF model, 32 SNPs are identified and selected for the disease at a 95% confidence level for the ADNI dataset. Besides, 36 SNPs for the GenADA and 218 SNPs for the NCRAD datasets were associated with the disease at a 99% confidence level (Supplementary Table 1). These prioritized SNPs are used for examining the 3-way interactions related to the LOAD.

### 3-Way interaction information analysis for prioritizing triplets

Selected SNPs from the RF-RF model of each dataset have been used to determine the significant three-way interactions. First, the rate of prioritized genotype triplets is calculated separately in case and control groups for each dataset. Then, case and control groups estimate the difference of each variant triplet’s three-way interaction (3WI) information. For the GenADA dataset, nine triplets had significant three-way interactions. However, one of the triplets consisting of rs17067596, rs4895529, and rs16993582 is filtered out since it includes an SNP pair in strong linkage disequilibrium. Likewise, ADNI and NCRAD datasets have 17 and 86 significantly interacting triplets.

In the next step, two-way mutual information gain is calculated for the variants found in the significant triplets. The SNP triplets with SNP combinations with significant two-way mutual information gain are excluded. For GenADA and ADNI, no SNP pairs with significant two-way mutual information gain are found. For NCRAD, 22 triplets with SNP pairs with significant two-way mutual information gain are filtered out (Supplementary Table 2). After this filtering, 8 significant triplets with 19 unique SNPs from GenADA, 17 with 26 unique SNPs from ADNI, and 64 significant triplets with 116 unique SNPs from the NCRAD dataset are prioritized. There were no common SNPs between these groups.

Lastly, for the validation of *p*-values assigned by test statistics, a permutation test was performed. Disease status labels were randomly shuffled, and information gain-based test statistic was calculated for the resulting triplets in each iteration as described in the methods. Lastly, 1000 permutations were performed for eight GenADA significant triplets and 17 ADNI significant triplets. 10,000 permutations for 64 NCRAD significant triplets as NCRAD was a larger genotyping platform. After permutation testing, significant triplets with a *p*-value lower than 0.05 are selected for the GenADA and ADNI datasets. Since there were more triplets for the NCRAD dataset, 0.01 was used as the *p*-value threshold.

### GenADA triplets

**Fig. 2:**
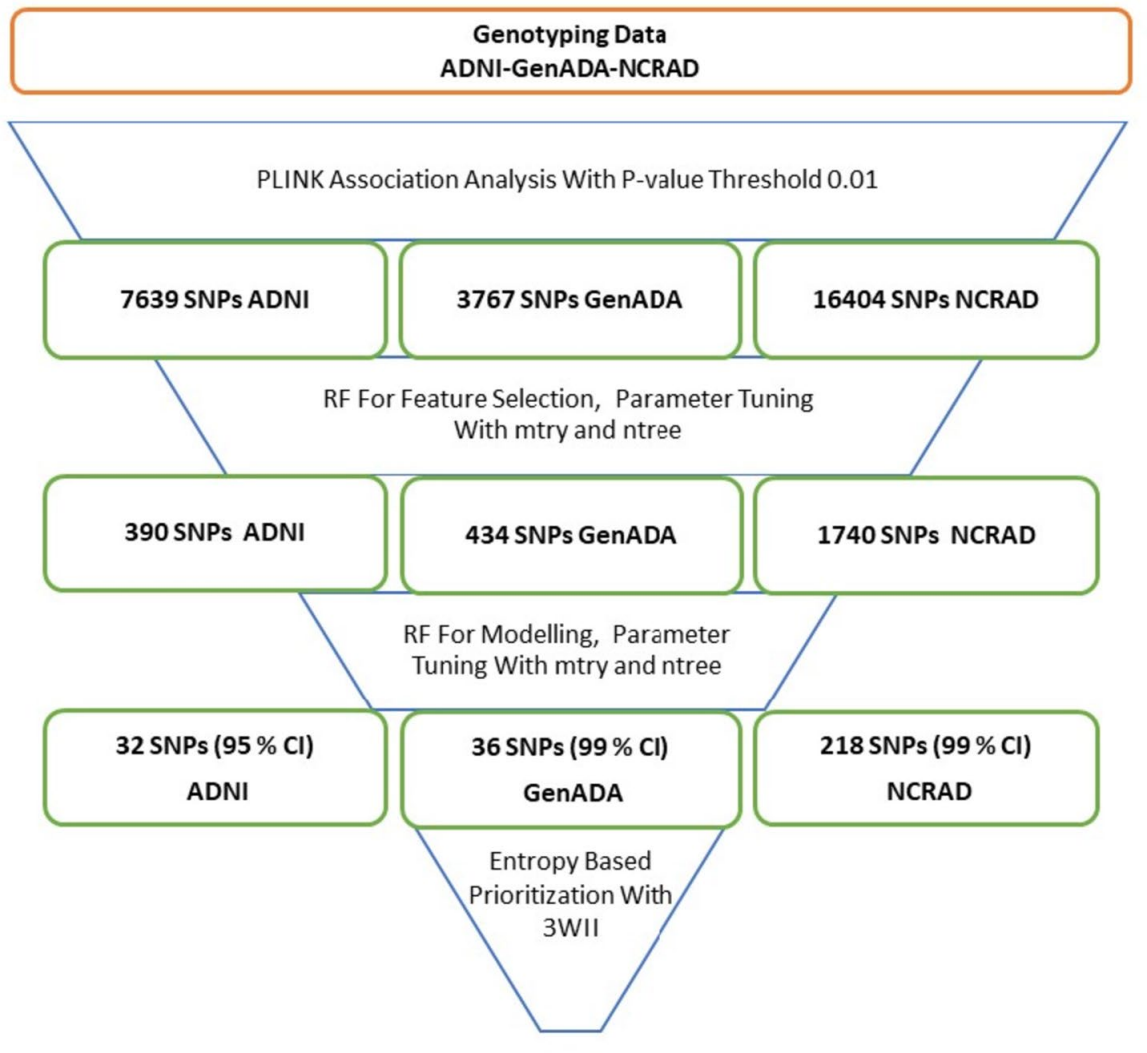
Workflow and data summary: in the first step, PLINK association analysis is performed for genotyping data of three datasets. Then RF-RF is conducted for feature selection and modeling, respectively. Output variants of the PLINK-RF-RF model are prioritized by 3WII analysis

Following the workflow summarized in Fig. 2, eight triplets with 19 unique SNPs for GenADA had significant 3-way interaction (3WI) information (Table 1). All GenADA 3WII SNPs are categorized as modifiers based on their impact on the SnpEff tool [49]. Four SNPs are mapped to FBLN2, ADAM 10, NHSL1, and ST3GAL1 genes previously associated with Alzheimer Disease [50, 51]. The SNP mapped to ADAM10 is also associated with the reticulate acropigmentation of Kitamur. Lastly, one variant is mapped to the RUNX1 gene associated with chronic myeloid leukemia (Table 2).

**Table 1:** Test statistics and permutation testing results for GenADA dataset

**SNP1**	**SNP2**	**SNP3**	**T**_**IG**_	***P*-value**	**Permutation *p*-value**	**Gene1**	**Gene2**	**Gene3**
rs17793957	rs605928	rs9911460	8.50	0.003	0.001	FBLN2	ADAM10	NPLOC4
rs7045548	rs1795977	rs11652714	8.25	0.004	0.001	-	-	-
rs1879019	rs17081694	rs605928	6.65	0.009	0.002	ADAM10	-	-
rs1608169	rs11862388	rs16993582	7.14	0.007	0.003	-	-	RUNX1
rs4895529	rs9314604	rs17081694	8.47	0.003	0.006	NHSL1	ANGPT2 / MCPH1	-
rs17067596	rs9314604	rs17081694	8.46	0.003	0.008	NHSL1	-	
rs10050568	rs2978012	rs6098412	7.76	0.005	0.015	SPOCK1	ST3GAL1	-
rs1879019	rs1519959	rs136687	8.48	0.003	0.036	-	-	PHF21B

**Table 2:** The SNPs in significant triplets in GenADA dataset mapped to a gene associated with a disease

**rsID**	**Chr**	**Pos**	**Gene**	**Phenotype**
rs17067596	6	138,767,012	NHSL1	Alzheimer Disease
rs2978012	8	134,539,196	ST3GAL1	Alzheimer Disease
rs4895529	6	138,770,275	NHSL1	Alzheimer Disease
r605928	15	59,046,163	ADAM10	Alzheimer Disease/ Reticulate acropigmentation of Kitamura
rs17793957	3	13,649,920	FBLN2	Alzheimer Diesase
rs9314604	8	6,411,499	MCPH1/ ANGPT2	Microcephaly
rs16993582	21	37,110,209	RUNX1	Platelet disorder, familial, with associated myeloid malignancy

Also, we used the direction of the IIG value representing the difference between the 3-way interaction information of disease and control groups as a marker for disease risk. A positive IIG represents the gain of 3WII in the presence of a disease. In contrast, a negative IIG represents the gain of 3WII in the general population versus the affected population. In the GenADA dataset for only two triplets, rs1608169; rs11862388; rs16993582, and rs10050568; rs2978012; rs6098412, the IIG was negative, suggesting increased risk, and it was positive for the rest of the triplets suggesting a protective effect (Supplementary Table 3).

### ADNI triplets

Seventeen triplets with 27 unique SNPs showed significant 3-way interaction (3WI) information after permutation testing identified for the ADNI Dataset (Table 3). One variant mapped to FERMT2 is revealed as a risk factor for Alzheimer disease. Four SNPs mapped to SYCP2L, VAV, SEPSECS, and TMPRSS15 are known to be associated with age-related hearing impairment, multiple sclerosis, pontocerebellar hypoplasia type 2, enterokinase deficiency, respectively. PLAGL1 is associated with transient neonatal diabetes mellitus and paternal uniparental disomy of chromosome 6, and NPSR1 is associated with asthma-related traits (Table 4).

**Table 3:** Test statistics and permutation testing results for ADNI dataset

**SNP1**	**SNP2**	**SNP3**	T_IG_	***P*-value**	**Permutation *p*-value**	**Gene1**	**Gene2**	**Gene3**
rs9366664	rs3780792	rs1150360	8.53	0.003	0.001	SYCP2L	VAV2	FAM76B
rs6705017	rs10017010	rs557098	7.51	0.006	0.001	UGGT1	PI4K2B	ALDH3B1
rs11749731	rs3780792	rs7157639	6.74	0.009	0.001	NDFIP1	VAV2	FERMT2
rs1023276	rs324389	rs2824808	8.81	0.002	0.002	-	NPSR1-AS1	TMPRSS15
rs6751810	rs4561856	rs1023276	7.92	0.004	0.002	-	-	-
rs4561856	rs4409091	rs2633466	7.61	0.005	0.002	-	-	-
rs4561856	rs10807701	rs2824808	7.55	0.005	0.002	-	TPST1	TMPRSS15
rs7091014	rs11006011	rs2633466	7.47	0.006	0.002	-	-	-
rs6856771	rs7157639	rs2824808	8.18	0.004	0.003	-	FERMT2	TMPRSS15
rs9366664	rs10960174	rs1150360	7.69	0.005	0.003	SYCP2L	-	FAM76B
rs11749731	rs10807701	rs7157639	7.42	0.006	0.003	NDFIP1	TPST1	FERMT2
rs6705017	rs11006011	rs2633466	7.77	0.005	0.005	UGGT1	-	-
rs9313264	rs12056012	rs2633466	7.69	0.005	0.005	-	-	-
rs6705017	rs2633466	rs462074	7.38	0.006	0.006	UGGT1	-	-
rs10017010	rs9313264	rs2207851	8.65	0.003	0.008	PI4K2B	-	PLAGL1
rs4561856	rs9896368	rs2824808	8.88	0.002	0.009	-	MMP28	TMPRSS15
rs4561856	rs7157639	rs717840	7.784	0.005	0.022	-	FERMT2	CDH13

**Table 4:** The SNPs in significant triplets in ADNI dataset mapped to a gene associated with a disease

**rsID**	**Chr**	**Pos**	**Gene**	**Phenotype**
rs7157639	14	53,388,161	FERMT2	Alzheimer Disease/Hereditary Spastic Paraplegia
rs9366664	6	10,892,499	SYCP2L	Age-related hearing impairment
rs10017010	4	25,188,718	SEPSECS	Pontocerebellar hypoplasia type 2es
rs2207851	6	144,337,886	PLAGL1	Transient neonatal diabetes mellitus / Paternal uniparental disomy of chromosome 6
rs2824808	21	19,775,220	TMPRSS15	Enterokinase deficiency
rs324389	7	34,777,714	NPSR1	Asthma-Related Traits
rs3780792	9	136,835,343	VAV2	Multiple Sclerosis

Although some triplets have common SNP pairs, they were not linked. All these SNPs are also categorized as modifiers. Additionally, in this dataset, only two triplets (rs6705017; rs10017010; rs557098 and rs685677; rs7157639; rs2824808) had negative IIG, suggesting increased risk for LOAD (Supplementary Table 4).

### NCRAD triplets

Fifty-two triplets with 106 unique SNPs were significant 3WI for NCRAD (Supplementary Table 5). Two triplets shared a common SNP pair. The third SNPs of the triplets, rs12663008 and rs17830067, were in the same LD region. So, one of these triplets could be used as a representative. There were also three other SNPs in the same LD region, which do not interact with other common SNPs.

Several variants mapped to PVRL2, TOMM40, LCMT1, and RAB3GAP1 genes previously associated with Alzheimer disease. Besides, another variant mapped to HNRNPA1 is associated with amyotrophic lateral sclerosis and inclusion body myopathy with Paget’s disease of bone and frontotemporal dementia (Table 5).

**Table 5:** The SNPs in NCRAD dataset mapped to a gene associated with a disease

**rsID**	**Chr**	**Pos**	**Gene**	**Phenotype**
rs2075650	19	45,395,619	TOMM40	Alzheimer Disease
rs6859	19	45,382,034	PVRL2	Alzheimer Disease
rs10445686	2	135,893,372	RAB3GAP1	Alzheimer Disease/Leiomyoma, Uterine
rs11645986	16	25,127,645	LCMT1	Alzheimer Disease
rs1920045	12	54,670,398	HNRNPA1	Amyotrophic lateral sclerosis/ inclusion body myopathy with Paget disease of bone and frontotemporal dementia
rs7181139	15	77,977,667	LINGO1	Mental retardation / Essential tremor & Parkinson's
rs3775162	4	72,397,710	SLC4A4	Proximal renal tubular acidosis with ocular abnormalities
rs4076290	2	1,378,969	TPO	Thyroid dyshormonogenesis
rs1560964	15	33,766,809	RYR3	Epileptic encephalopathy
rs1530498	5	13,902,220	DNAH5	Primary ciliary dyskinesia
rs17576289	3	45,458,733	LARS2	Perrault syndrome
rs17742907	22	18,890,615	DGCR6	Velocardiofacial syndrome
rs2108392	5	130,533,828	LYRM7	Mitochondrial Complex iii Deficiency
rs2432762	6	5,435,756	FARS2	Combined oxidative phosphorylation defect type 14
rs3785113	16	68,369,213	PRMT7	Pseudohypoparathyroidism-like disorder
rs3888795	18	11,863,899	GNAL	Dystonia
rs991974	6	70,481,267	LMBRD1	Methylmalonic acidemia with homocystinuria

The SNPs are categorized as modifiers as in the other dataset groups. However, unlike the other datasets, IIG values are mostly positive in NCRAD. Only 14 triplets are positive, suggesting protective effect.

### Functional enrichment

Functional enrichment analysis was conducted for the gene set, which combined the genes that variants mapped in all three datasets. These variants are annotated with SNPNexus. The functional enrichment analysis involves overlapped, nearest upstream and downstream genes (Supplementary Table 6).

Firstly, GO Molecular Function, GO Cellular Component, GO Biological Process, and Reactome pathways are obtained for the resulting dataset. Calcium ion binding, extracellular matrix, external encapsulating structure, and RUNX1 regulates estrogen receptor-mediated transcription pathways are significantly enriched.

**Fig. 3:**
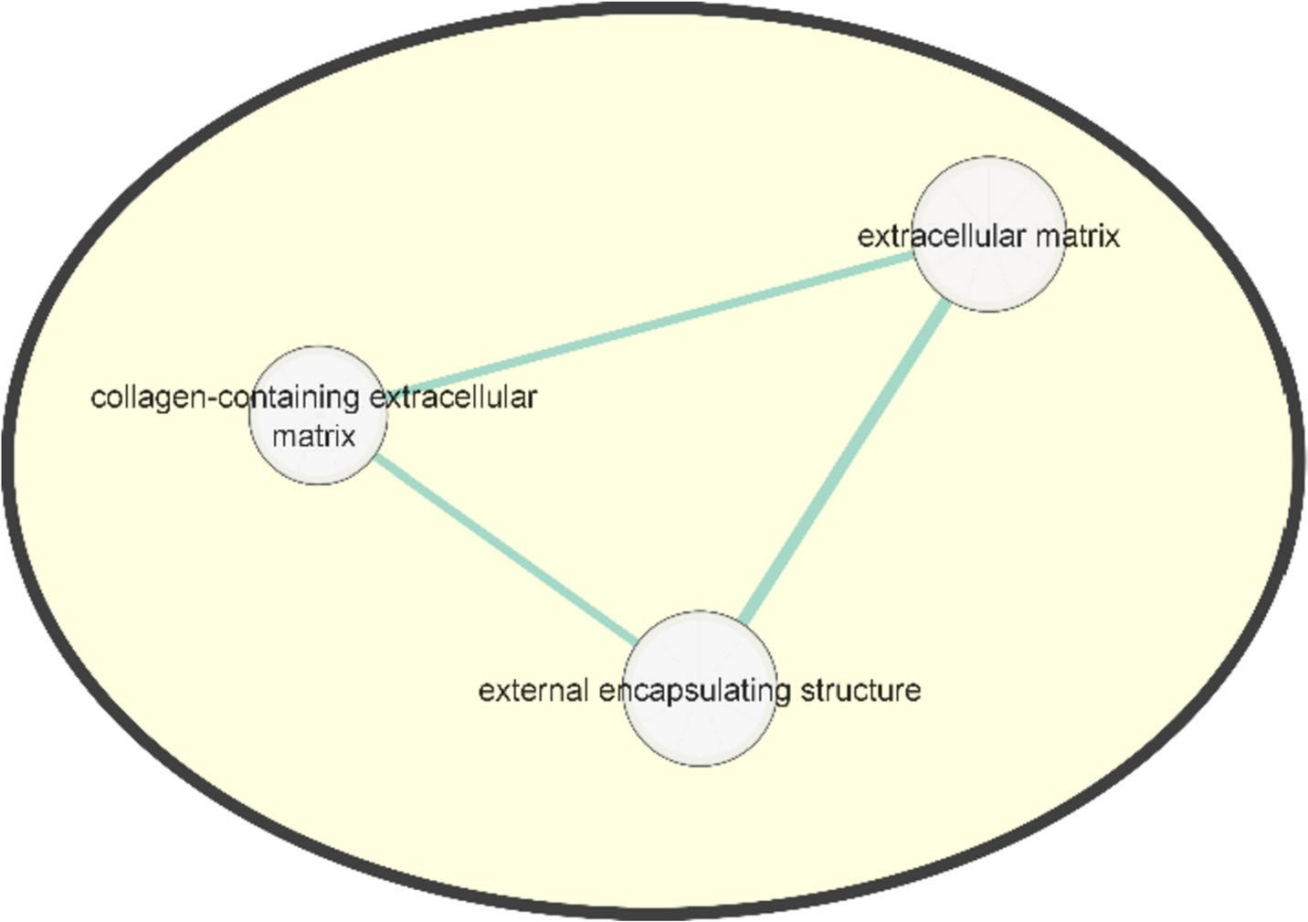
Functional enrichment network created by Enrichment Map

Then, functional enrichment networks are created by Enrichment Map. The common functions of the extracellular matrix and external encapsulating structure pathways are observed on the same network (Fig. 3).

## Discussion

Like other complex diseases, unveiling the missing heritability is challenging in AD. Various studies have revealed epistatic relationships and offer a potential solution for this problem. In earlier studies, interactions between genes in pathways associated with AD are examined [52, 53], while recent studies focus on the interactions between GWAS-identified LOAD genes [54]. Also, few studies reported epistatic effects associated with AD’s endophenotype or intermediate traits, such as amyloid deposition and brain atrophy [14, 29, 30].

The limitations of epistatic interaction studies are the extensive multiple comparisons and low power, which give rise to high error rates of type 1 and type 2. Besides, the high cost of computational resources required for an exhaustive search is another constraint that makes detecting the interactions challenging. These limitations also avoid producing replicated results. Therefore, we have proposed an integrative approach with the multi-step RF-RF for model minimization and 3WII to reveal epistatic interactions overcoming the computational limitations. The proposed LOAD-RF-RF model is developed using PLINK and RF-RF workflow for three LOAD datasets from different datasets. Considering the utilization of a large number of SNPs in our study, we acknowledge the significance of discussing potential challenges associated with high-dimensional data. High dimensionality can introduce complexity to the model, making it more prone to overfitting and decreasing interpretability. We have addressed this by employing feature selection, cross-validation, and parameter tuning to ensure our model generalizes well to new data and retains its predictive accuracy.

We employed permutation testing to validate significant interactions. While permutation testing is a robust hypothesis testing method, it comes with computational challenges, particularly when applied to large datasets. We are still exploring alternative statistical approaches and considerations to deliver efficient and dependable results. These alternative methods might encompass approximation techniques, resampling strategies, or customized variations of permutation testing, carefully crafted to alleviate computational demands while upholding the essential standards of statistical rigor.

Then, triplets with significant 3-way interactions are identified among the prioritized SNPs. RF is a powerful tool to address the missing heritability problem as it reveals high dimensional interactions between variants. Implementing the 3WII step allowed us to recognize the informative variant triplets among the LOAD-RF-RF prioritized SNPs, which could be informative candidate biomarkers. Conversely, Hohman et al. and Gusareva et al. also performed an exhaustive genome-wide interaction information analysis [55, 56]. Hohman et al. used a biological knowledge-driven approach to assess the interactions, while we propose a model minimization approach instead of using the bias-prone prior knowledge approach to reveal the significant interactions. Apart from that, both prior studies focus on 2-way rather than 3-way interactions.

For all three datasets, the number of unique SNPs is reduced after the 3WII analysis, and it was promising to apply 3-way interaction information analysis for model minimization. Similarly, prioritization of SNPs through multi-step RF before 3-WII analysis reduces the need for extensive computational resources for exhaustive analysis.

3WII analysis is performed to prioritize SNPs by LOAD-RF-RF model for all datasets. As the number of SNPs prioritized differs between different datasets, the number of significant triplets differed. Also, we observed more significant interactions with the NCRAD dataset, which was the largest dataset. Although the NCRAD SNPs are reduced with the LOAD-RF-RF model before 3WII analysis like the other two datasets, more SNPs are prioritized, and more significant triplets are identified.

Our study involves a limited number of subjects, and we acknowledge that larger sample sizes are generally desirable for maintaining statistical power, but there are specific factors to consider in our case. The availability of certain datasets, like the UK genomics dataset, was restricted, and we had to rely on datasets to which we had controlled access. These were the most extensive datasets available to us for the scope of our research. Despite the sample size limitations, it is worth noting that our study design and analysis workflow have inherent strengths. The proposed triple-based approach offers an advantage in identifying relationships beyond what statistical power alone can achieve. It allows us to explore connections between variables that may not be apparent in traditional large-sample GWAS studies.

Complex diseases have polygenic etiology since multiple genes and environmental interactions contribute to the phenotype. We have observed that most common disease-risk variants map to noncoding sequences, known as modifiers, as expected. Also, in literature, complex disease genes overlap with genes related to Mendelian disorders. Our observations are parallel with the literature as some significant interactions revealed for LOAD are SNPs mapped to Mendelian disorder genes. Up to ten variants are mapped to genes associated with Alzheimer Disease within the prioritized SNPs in all datasets. Around five SNPs are associated with neurological disorders like multiple sclerosis, epileptic encephalopathy, and Parkinson’s disease. 3-way interaction information describes the information gained for all variables but does not present any subset alone. Therefore, although prioritized SNPs are not previously annotated as LOAD-associated SNPs, their interaction could still inform about the LOAD risk.

IIGs are calculated for triplets dependent on the disease based on test statistics (T_IG_). Only a few IIGs are positive for GenADA and ADNI datasets. IIGs for 38 triplets out of 52 significant triplets have positive IIG in the NCRAD dataset. A positive IIG indicates that the disease group’s 3-way interaction information is greater than the control group. The IIG-positive triplets are proposed as risk variants for LOAD, while the IIG-negative triplets should be further investigated as protective markers. Further clinical studies can validate the prioritized SNPs and triplets’ association with the LOAD.

Our study employed reputable annotation tools, SNPNexus and SNiPA, to annotate genomic variants. These tools have been widely recognized for their utility in providing valuable insights into the functional implications of genetic variants. However, it is essential to acknowledge that no annotation tool can comprehensively encompass all genomic variants, and therefore, their coverage may have inherent limitations. It is crucial to be aware of these limitations when interpreting the results.

Functional enrichment analysis of 3WI variants from three different LOAD datasets at the gene level showed enrichment of collagen-containing extracellular matrix (ECM) and external encapsulating structure pathways. The ECM supports the basement membranes and microcircular environment of the tissues. In several recent studies, the link between changes in the ECM and aging and neurodegenerative diseases is reported [57, 58]. Even though the exact molecular impact of changes in the ECM proteins during AD development is still under investigation, its effects on synaptic transmission, amyloid-β-plaque generation and degradation, Tau-protein production, oxidative stress response, and inflammatory response have been reviewed [59].

Additionally, ERs have a role in cognition and memory. ERs also act as a neuroprotectant, modulating several neuroprotective pathways, immune response, neurogenesis, glial cell functions, and response to excitotoxicity. As the female predominance in developing AD suggests the involvement of gender-specific factor(s), the potential role of ER alpha in AD pathogenesis has been explored in many studies [60, 61].

These functional-level observations support the proposed entropy-based post-GWAS analysis, LOAD-RF-RF followed by 3WII, as the prioritized variants and genes show association with LOAD and provide insights into early LOAD pathogenesis. Nevertheless, although the variants of the prioritized triplets are not enriched in AD-associated functional pathways, their interactions can still imply the LOAD risk.

Besides, it is important to recognize that the interpretation of gene functions and pathways can vary depending on the specific context and the tools used. Our study employed the g:Profiler and EnrichmentMap as valuable resources. While these tools offer valuable insights, we acknowledge that the outcomes of such analyses may be context-dependent. In light of the potential limitations of specific tools and the context-dependent nature of gene function interpretation, alternative approaches such as utilizing different databases, algorithms, or statistical methods might be used. By considering and discussing alternative approaches, we aim to offer a more comprehensive view of the biological insights derived from our analysis.

We utilized Fisher’s exact test’s *p*-value in conjunction with the Bonferroni correction for multiple testing for the functional enrichment analysis. While these methods are established and widely used in statistical analysis, Fisher’s exact test assumes independence among observations as one limitation. Although we applied it with caution in situations where this assumption is reasonable, it is important to recognize that alternative tests that relax this assumption may be more appropriate in some cases. The Bonferroni correction is renowned for its conservative nature. While it effectively controls the familywise error rate, it does so by increasing the threshold for statistical significance. This can result in a higher risk of Type II errors, where true associations may go undetected. Also, the Bonferroni correction assumes independence among multiple tests. In cases where tests are correlated, as is often the case with multiple comparisons within a dataset, the correction can become overly stringent, potentially compromising statistical power. We are committed to addressing these limitations by considering the specific context of our research and the characteristics of our data. In cases where concerns about these limitations arise, we encourage researchers to explore alternative statistical methods and correction approaches that may be better suited to their research goals and data structure.

## Conclusion

Random forest and entropy-based methods reveal non-linear genetic and environmental factors contributing to complex traits. The proposed workflow in this study demonstrates an efficient framework for revealing the complex interactions that contribute significantly as genetic factors for LOAD. 3WII is used as a model minimization method while determining the significant 3-way interactions between the prioritized SNPs by PLINK-RF-RF. The SNPs detected by this optimized in-silico model could be examined in a clinical context to decide if the resulting triplets have predictive power for early or differential LOAD diagnosis.

This framework is a promising approach for post-GWAS analysis of other complex genetic disorders. The method can be improved by applying it to the GWAS data obtained from large-scale data repositories. It could also be modified by integrating other machine learning and entropy-based interaction methods.

### Supplementary Information

**Additional file 1: Supplementary Figure 1.** The optimized mtry and ntree parameters are specified as 2 and 900 for GenADA dataset1. **Supplementary Figure 2.** The model optimized with the mtry and ntree parameters in the ADNI dataset, by considering the diagnostic model error rate with the 5-folds cross-validation. Optimum mtry and optimum ntree parameters are specified as 39 and 50 respectively.** Supplementary Figure 3.** The optimized mtry and ntree parameters are specified as 83 and 1000 respectively for NCRAD dataset.** Supplementary Table 1.** SNPs Selected through PLINK-RF-RF workflow.** Supplementary Table 2.** Triplets that are filtered as they include SNP pairs with significant 2WI in the NCRAD dataset. **Supplementary Table 3.** IIG Values for Prioritized GenADA Triplets. **Supplementary Table 4.** IIG Values for Prioritized ADNI Triplets.** Supplementary Table 5.** Test Statistics and Permutation Testing Results for NCRAD Dataset.** Supplementary Table 6.** Genes involved in functional enrichment analysis.

## Data Availability

Both programming code and data are available upon request (yesim@metu.edu.tr).
