# Supplementary material for: Revealing third-order interactions through the integration of machine learning and entropy methods in genomic studies
Source: BioData Min. 2024 Jan 30;17:3. doi: 10.1186/s13040-024-00355-3 (PMC10826120; doi:10.1186/s13040-024-00355-3)
Supplement: Supplementary file 1 — Additional file 1: Supplementary Figure 1. The optimized mtry and ntree parameters are specified as 2 and 900 for GenADA dataset1. Supplementary Figure 2. The model optimized with the mtry and ntree parameters in the ADNI dataset, by considering the diagnostic model error rate with the 5-folds cross-validation. Optimum mtry and optimum ntree parameters are specified as 39 and 50 respectively. Supplementary Figure 3. The optimized mtry and ntree parameters are specified as 83 and 1000 respectively for NCRAD dataset. Supplementary Table 1. SNPs Selected through PLINK-RF-RF workflow. Supplementary Table 2. Triplets that are filtered as they include SNP pairs with significant 2WI in the NCRAD dataset. Supplementary Table 3. IIG Values for Prioritized GenADA Triplets. Supplementary Table 4. IIG Values for Prioritized ADNI Triplets. Supplementary Table 5. Test Statistics and Permutation Testing Results for NCRAD Dataset. Supplementary Table 6. Genes involved in functional enrichment analysis. [file 13040_2024_355_MOESM1_ESM.docx]

#
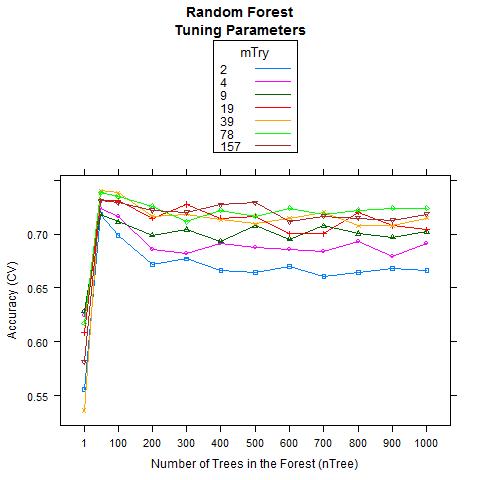
Supplementary Figures


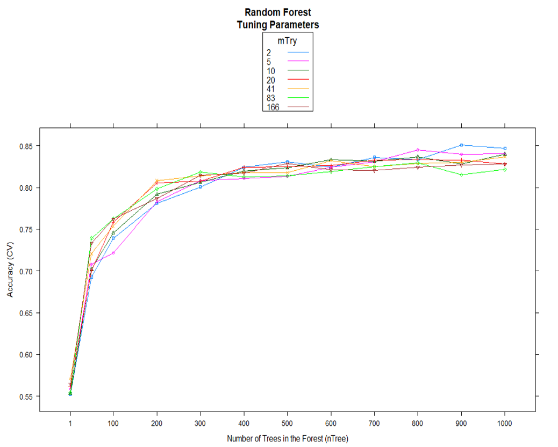


Supplementary Figure 1 The optimized mtry and ntree parameters are specified as 2 and 900 for GenADA dataset1

Supplementary Figure 2 The model optimized with the mtry and ntree parameters in the ADNI dataset, by considering the diagnostic model error rate with the 5-folds cross-validation. Optimum mtry and optimum ntree parameters are specified as 39 and 50 respectively

#
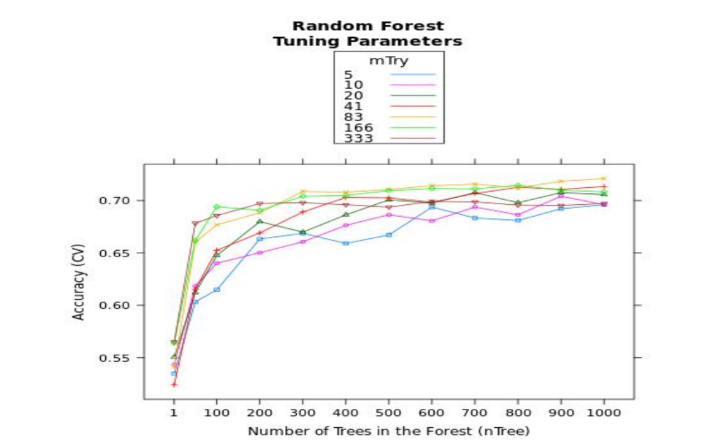


Supplementary Figure 3 The optimized mtry and ntree parameters are specified as 83 and 1000 respectively for NCRAD dataset

# Supplementary Tables

Supplementary Table 1 SNPs Selected through PLINK-RF-RF workflow

| ADNI | GenADA | NCRAD | | | | | | |
| --- | --- | --- | --- | --- | --- | --- | --- | --- |
| rs10017010 | rs2273570 | rs10022344 | rs12549680 | rs17519749 | rs2467261 | rs4760479 | rs7212762 | rs9957722 |
| rs2131006 | rs11810899 | rs1004677 | rs12663008 | rs17576289 | rs248471 | rs4770244 | rs7247886 | rs9966557 |
| rs557098 | rs1879019 | rs10056788 | rs12677921 | rs17742907 | rs2588478 | rs4774371 | rs7309474 | |
| rs6705017 | rs1553136 | rs10108019 | rs12683681 | rs17830067 | rs2781092 | rs4778582 | rs7314903 | |
| rs9307850 | rs17793957 | rs10240034 | rs12978451 | rs1793773 | rs2792752 | rs4789127 | rs7428284 | |
| rs9896368 | rs12493090 | rs1028723 | rs13030842 | rs1842076 | rs2821338 | rs4803586 | rs743026 |  |
| rs10788181 | rs16863803 | rs1033686 | rs13224531 | rs1872240 | rs2860001 | rs4841920 | rs7518469 | |
| rs10807701 | rs10024098 | rs10401458 | rs1322669 | rs1904616 | rs2953130 | rs495959 | rs7531902 | |
| rs11006011 | rs11743813 | rs10435761 | rs1333190 | rs1920045 | rs2966220 | rs4978508 | rs761009 |  |
| rs11749731 | rs10050568 | rs10445686 | rs13386681 | rs1920086 | rs3104398 | rs544166 | rs766787 |  |
| rs2824808 | rs17067596 | rs10458404 | rs13416635 | rs19334 | rs31726 | rs5752338 | rs7683253 | |
| rs6856771 | rs4895529 | rs10498554 | rs1372173 | rs2040761 | rs332847 | rs5989589 | rs7723920 | |
| rs7091014 | rs1834615 | rs10520536 | rs1382794 | rs2045896 | rs3744587 | rs6074067 | rs7726567 | |
| rs717840 | rs9314604 | rs10784286 | rs1407469 | rs2049969 | rs3753306 | rs6099457 | rs7743868 | |
| rs9313264 | rs11785331 | rs10804966 | rs1437635 | rs2060802 | rs3763696 | rs6102926 | rs7752524 | |
| rs2207851 | rs2978012 | rs10860105 | rs1439279 | rs2075650 | rs3775162 | rs6151630 | rs7761785 | |
| rs462074 | rs4073002 | rs10877627 | rs1447830 | rs2088746 | rs3777171 | rs6449986 | rs7769144 | |
| rs6751810 | rs7045548 | rs10877894 | rs1450913 | rs2105806 | rs3785113 | rs6455400 | rs7876155 | |
| rs12056012 | rs10795618 | rs10922743 | rs1459241 | rs2108392 | rs3790630 | rs6455403 | rs7971558 | |
| rs3967101 | rs472186 | rs10941112 | rs1489449 | rs2118844 | rs3806448 | rs6474161 | rs8030257 | |
| rs4954943 | rs1608169 | rs10945826 | rs1530498 | rs2151641 | rs3816620 | rs6480813 | rs837957 |  |
| rs7157639 | rs1795977 | rs11015383 | rs1552673 | rs2166001 | rs3827466 | rs6494944 | rs839933 |  |
| rs9366664 | rs1519959 | rs11023775 | rs1558139 | rs2223541 | rs3843725 | rs6506593 | rs8802 |  |
| rs1023276 | rs17081694 | rs11049103 | rs1560964 | rs2244263 | rs3888795 | rs6604921 | rs882299 |  |
| rs10960174 | rs605928 | rs11049111 | rs16822383 | rs2256331 | rs4076290 | rs6645551 | rs920633 |  |
| rs1150360 | rs2028389 | rs11235796 | rs16826325 | rs2268408 | rs4255200 | rs6743559 | rs931774 |  |
| rs2548032 | rs7204799 | rs1149938 | rs16841113 | rs2283821 | rs4307816 | rs6789329 | rs9367597 | |
| rs2633466 | rs11862388 | rs11645986 | rs16841178 | rs2297741 | rs4450019 | rs6789642 | rs943724 |  |
| rs324389 | rs11652714 | rs11782215 | rs16861648 | rs2299776 | rs4478858 | rs6859 | rs9454454 | |
| rs3780792 | rs9911460 | rs11889431 | rs16863430 | rs2301718 | rs4498832 | rs6887169 | rs9490248 | |
| rs4409091 | rs52911 | rs11924077 | rs16985682 | rs2310878 | rs4535265 | rs6904295 | rs9516391 | |
| rs4561856 | rs10402361 | rs12135108 | rs17043361 | rs2325652 | rs4634829 | rs6942330 | rs9580739 | |
|  | rs12974182 | rs12236795 | rs17080902 | rs2385507 | rs4648489 | rs6948502 | rs961848 |  |
|  | rs6098412 | rs12379827 | rs17148940 | rs2420516 | rs4668565 | rs6990287 | rs9670249 | |
|  | rs16993582 | rs12468084 | rs17149283 | rs2432762 | rs4750306 | rs7110148 | rs9691167 | |
|  | rs136687 | rs12483892 | rs17247661 | rs2436111 | rs4755903 | rs7181139 | rs991974 |  |

Supplementary Table 2 Triplets that are filtered as they include SNP pairs with significant 2WI in the NCRAD dataset

| SNP1 | SNP2 | SNP3 | TIG | p-value |
| --- | --- | --- | --- | --- |
| rs1004677 | rs4770244 | rs9966557 | 10.96 | 0.0009 |
| rs10445686 | rs11924077 | rs16822383 | 11.14 | 0.0008 |
| rs10458404 | rs11924077 | rs839933 | 10.89 | 0.001 |
| rs11924077 | rs11235796 | rs9670249 | 11.37 | 0.0007 |
| rs11924077 | rs7110148 | rs9670249 | 11.68 | 0.0006 |
| rs11924077 | rs839933 | rs10498554 | 12.08 | 0.0005 |
| rs13416635 | rs1028723 | rs3888795 | 12.71 | 0.0004 |
| rs13416635 | rs1028723 | rs9966557 | 11.84 | 0.0006 |
| rs13416635 | rs1028723 | rs16985682 | 11.69 | 0.0006 |
| rs13416635 | rs11924077 | rs839933 | 11.04 | 0.0009 |
| rs13416635 | rs11924077 | rs10498554 | 14.52 | 0.0001 |
| rs13416635 | rs4770244 | rs9966557 | 11.90 | 0.0006 |
| rs16822383 | rs11023775 | rs1028723 | 10.98 | 0.0009 |
| rs16822383 | rs12663008 | rs17830067 | 11.82 | 0.0006 |
| rs2821338 | rs6942330 | rs9580739 | 10.86 | 0.001 |
| rs3775162 | rs9580739 | rs9966557 | 10.84 | 0.001 |
| rs3806448 | rs991974 | rs16985682 | 13.60 | 0.0002 |
| rs4450019 | rs991974 | rs16985682 | 12.16 | 0.0005 |
| rs4770244 | rs9580739 | rs9966557 | 12.04 | 0.0005 |
| rs6449986 | rs9670249 | rs3888795 | 11.49 | 0.0007 |
| rs9580739 | rs1028723 | rs16985682 | 11.87 | 0.0005 |
| rs991974 | rs839933 | rs16985682 | 11.49 | 0.0007 |

Supplementary Table 3 IIG Values for Prioritized GenADA Triplets

| SNP1 | SNP2 | SNP3 | IIG |
| --- | --- | --- | --- |
| rs17793957 | rs605928 | rs9911460 | 0.02 |
| rs7045548 | rs1795977 | rs11652714 | 0.01 |
| rs1879019 | rs17081694 | rs605928 | 0.01 |
| rs1608169 | rs11862388 | rs16993582 | -0.1 |
| rs4895529 | rs9314604 | rs17081694 | 0.003 |
| rs17067596 | rs9314604 | rs17081694 | 0.003 |
| rs10050568 | rs2978012 | rs6098412 | -0.01 |
| rs1879019 | rs1519959 | rs136687 | 0.01 |

Supplementary Table 4 IIG Values for Prioritized ADNI Triplets

| **SNP1** | **SNP2** | **SNP3** | **IIG** |
| --- | --- | --- | --- |
| rs9366664 | rs3780792 | rs1150360 | 0.07 |
| rs6705017 | rs10017010 | rs557098 | -0.01 |
| rs11749731 | rs3780792 | rs7157639 | 0.05 |
| rs1023276 | rs324389 | rs2824808 | 0.08 |
| rs6751810 | rs4561856 | rs1023276 | 0.05 |
| rs4561856 | rs4409091 | rs2633466 | 0.07 |
| rs4561856 | rs10807701 | rs2824808 | 0.06 |
| rs7091014 | rs11006011 | rs2633466 | 0.1 |
| rs6856771 | rs7157639 | rs2824808 | -0.03 |
| rs9366664 | rs10960174 | rs1150360 | 0.07 |
| rs11749731 | rs10807701 | rs7157639 | 0.04 |
| rs6705017 | rs11006011 | rs2633466 | 0.05 |
| rs9313264 | rs12056012 | rs2633466 | 0.04 |
| rs6705017 | rs2633466 | rs462074 | 0.04 |
| rs10017010 | rs9313264 | rs2207851 | 0.05 |
| rs4561856 | rs9896368 | rs2824808 | 0.06 |
| rs4561856 | rs7157639 | rs717840 | 0.05 |

Supplementary Table 5 Test Statistics and Permutation Testing Results for NCRAD Dataset

| SNP1 | SNP2 | SNP3 | TIG | p-value | Permuation p-value | Gene1 | Gene2 | Gene3 | IIG |
| --- | --- | --- | --- | --- | --- | --- | --- | --- | --- |
| rs10056788 | rs10108019 | rs931774 | 12.67 | 0.0003 | 0.001 | NIM1K | ADAM18 | SOX6 | -0.009 |
| rs11924077 | rs2108392 | rs7971558 | 12.21 | 0.0004 | 0.001 | AADACL2-AS1 | LYRM7 | REP15 | -0.004 |
| rs12379827 | rs837957 | rs11645986 | 10.88 | 0.0009 | 0.001 |  |  | LCMT1 | -0.01 |
| rs12468084 | rs2420516 | rs3763696 | 10.92 | 0.0009 | 0.001 |  |  |  | -0.01 |
| rs12663008 | rs7247886 | rs4634829 | 13.63 | 0.0002 | 0.001 |  |  |  | 0.01 |
| rs17043361 | rs13030842 | rs7309474 | 11.12 | 0.0008 | 0.001 | DPP10 | RAPGEF4 |  | 0.009 |
| rs17148940 | rs2297741 | rs4307816 | 10.97 | 0.0009 | 0.001 | ZNF474 | LAMA2 |  | -0.006 |
| rs17830067 | rs7247886 | rs4634829 | 13.73 | 0.0002 | 0.001 |  |  |  | 0.01 |
| rs19334 | rs11023775 | rs2299776 | 11.40 | 0.0007 | 0.001 | PPP1R3B |  | PCP4 | 0.01 |
| rs2385507 | rs12379827 | rs3888795 | 17.15 | 3.45E-05 | 0.001 | ANXA13 |  | GNAL | -0.02 |
| rs2420516 | rs1560964 | rs3888795 | 10.89 | 0.0009 | 0.001 |  | RYR3 | GNAL | 0.01 |
| rs2860001 | rs12683681 | rs17742907 | 12.15 | 0.0004 | 0.001 |  | C9orf84 | DGCR6 | -0.01 |
| rs2953130 | rs2432762 | rs12379827 | 12.48 | 0.0004 | 0.001 |  | FARS2 |  | -0.01 |
| rs4076290 | rs3775162 | rs2060802 | 12.60 | 0.0003 | 0.001 | TPO | SLC4A4 | ADAM18 | -0.02 |
| rs6474161 | rs839933 | rs9966557 | 14.41 | 0.0001 | 0.001 | ADAM18 |  |  | -0.01 |
| rs6789329 | rs7761785 | rs4978508 | 12.58 | 0.0003 | 0.001 |  | HCRTR2 | SNX30 | -0.02 |
| rs9367597 | rs1028723 | rs4803586 | 11.11 | 0.0008 | 0.001 | FAM83B | CHORDC2P | PSG4 | 0.01 |
| rs10108019 | rs839933 | rs9966557 | 14.98 | 0.0001 | 0.002 | ADAM18 |  |  | -0.01 |
| rs11924077 | rs7314903 | rs9957722 | 15.08 | 0.0001 | 0.002 | AADACL2-AS1 | FAM19A2 | RNA5SP458 | -0.01 |
| rs11924077 | rs9454454 | rs31726 | 12.40 | 0.0004 | 0.002 | AADACL2-AS1 | FGF12 | PLEKHG2 | -0.01 |
| rs13224531 | rs6990287 | rs1149938 | 12.65 | 0.0004 | 0.002 |  |  |  | -0.01 |
| rs17080902 | rs17148940 | rs991974 | 12.61 | 3.82E-04 | 0.002 |  | ZNF474 | LMBRD1 | -0.005 |
| rs2108392 | rs2792752 | rs3785113 | 10.88 | 0.001 | 0.002 | LYRM7 | GJC1 | PRMT7 | -0.009 |
| rs2268408 | rs3763696 | rs10860105 | 11.26 | 0.0008 | 0.002 | MDFI |  | NEDD1 | -0.02 |
| rs2301718 | rs2420516 | rs839933 | 12.40 | 0.0004 | 0.002 |  |  |  | -0.01 |
| rs3753306 | rs2045896 | rs6480813 | 11.55 | 0.0007 | 0.002 | SELP | C1orf112 | C10orf11 | -0.01 |
| rs4535265 | rs1004677 | rs839933 | 12.34 | 0.0004 | 0.002 | FYCO1 | LINC-PINT |  | -0.01 |
| rs4535265 | rs1920045 | rs4760479 | 11.64 | 0.0006 | 0.002 | FYCO1 | CBX5/ HNRNPA1 |  | 0.02 |
| rs6887169 | rs10108019 | rs931774 | 12.42 | 0.0004 | 0.002 | NIM1K | ADAM18 | SOX6 | -0.01 |
| rs6904295 | rs7110148 | rs1920086 | 11.11 | 0.000858 | 0.002 |  | SLC6A5 | FAM19A2 | 0.01 |
| rs7428284 | rs2953130 | rs4770244 | 12.42 | 0.0004 | 0.002 | ROBO2 |  | MTND3P1 | -0.01 |
| rs10804966 | rs9966557 | rs332847 | 11.60 | 0.0006 | 0.003 | EVC |  | SIPA1L3 | -0.001 |
| rs16841178 | rs7683253 | rs5989589 | 17.80 | 2.45E-05 | 0.003 |  |  |  | 0.01 |
| rs16863430 | rs11049103 | rs6859 | 11.02 | 0.0009 | 0.003 |  | MRPS35 | PVRL2 | -0.02 |
| rs16863430 | rs991974 | rs31726 | 12.27 | 0.0005 | 0.003 |  | LMBRD1 | PLEKHG2 | -0.01 |
| rs2420516 | rs7726567 | rs1920086 | 12.19 | 0.0005 | 0.003 |  |  | FAM19A2 | -0.01 |
| rs6455403 | rs6990287 | rs7876155 | 11.64 | 0.0006 | 0.003 |  |  | FRMPD4 | 0.01 |
| rs7181139 | rs2966220 | rs2223541 | 11.65 | 0.0006 | 0.003 | LINGO1 |  | PTPRT | 0.01 |
| rs16822383 | rs19334 | rs12978451 | 11.81 | 0.0006 | 0.004 |  | PPP1R3B | ZNF331 | -0.01 |
| rs10941112 | rs4307816 | rs1028723 | 11.66 | 6.38E-04 | 0.005 | AMACR |  | CHORDC2P | -0.01 |
| rs9490248 | rs931774 | rs1033686 | 11.04 | 0.0009 | 0.005 |  | SOX6 | RAD51B | -0.01 |
| rs10445686 | rs17576289 | rs2075650 | 11.90 | 0.0006 | 0.006 | RAB3GAP1/ SNORA40/ ZRANB3 | LARS2 | TOMM40 | 0.02 |
| rs17148940 | rs1552673 | rs16985682 | 11.90 | 0.0006 | 0.006 | ZNF474 | ARRDC4 |  | -0.01 |
| rs1842076 | rs12683681 | rs17742907 | 12.15 | 0.0005 | 0.006 | - | C9orf84 | DGCR6 | -0.01 |
| rs7743868 | rs6990287 | rs7876155 | 11.09 | 0.0009 | 0.006 |  |  | FRMPD4 | 0.01 |
| rs9490248 | rs1437635 | rs1033686 | 11.69 | 0.0006 | 0.006 |  | SOX6 | RAD51B | -0.01 |
| rs11889431 | rs6887169 | rs3763696 | 12.10 | 0.0005 | 0.009 | THSD7B | NIM1K |  | -0.01 |
| rs13386681 | rs7181139 | rs9957722 | 11.91 | 0.0005 | 0.009 | ATOH8 | LINGO1 | RNA5SP458 | -0.01 |
| rs16861648 | rs3888795 | rs9966557 | 11.18 | 0.0008 | 0.009 | IGSF21 | GNAL |  | 0.01 |
| rs17080902 | rs9580739 | rs7181139 | 13.60 | 0.0002 | 0.009 |  |  | LINGO1 | -0.01 |
| rs2860001 | rs9580739 | rs6102926 | 15.13 | 0.0001 | 0.009 |  |  | PTPRT | -0.01 |
| rs495959 | rs7181139 | rs12483892 | 11.79 | 0.0006 | 0.009 | SLC6A17 | LINGO1 |  | -0.01 |
| rs17080902 | rs9670249 | rs10401458 | 11.21 | 0.0008 | 0.013 | SPTBN4 |  |  | -0.01 |
| rs2060802 | rs7181139 | rs3785113 | 11.77 | 0.0006 | 0.016 | ADAM18 | PRMT7 | LINGO1 | -0.01 |
| rs13386681 | rs7181139 | rs3888795 | 13.98 | 0.0002 | 0.018 | ATOH8 | GNAL | LINGO1 | -0.01 |
| rs16841113 | rs10520536 | rs9516391 | 12.06 | 0.0005 | 0.018 | TENM3 |  |  | -0.01 |
| rs4478858 | rs6474161 | rs7181139 | 10.9 | 0.001 | 0.018 | SERINC2 | ADAM18 | LINGO1 | -0.01 |
| rs16841113 | rs10520536 | rs4307816 | 12.4 | 0.0004 | 0.02 | TENM3 |  |  | -0.01 |
| rs9670249 | rs7247886 | rs12483892 | 14.03 | 0.0001 | 0.021 |  |  |  | -0.01 |
| rs12135108 | rs991974 | rs7181139 | 11.05 | 0.0009 | 0.022 | MLK4 | LINGO1 | LMBRD1 | -0.01 |
| rs17519749 | rs6474161 | rs7181139 | 11.09 | 0.0009 | 0.022 | ADAM18 | LINGO1 |  | -0.01 |
| rs7428284 | rs1530498 | rs4255200 | 11.37 | 0.0007 | 0.022 | DNAH5 | ROBO2 |  | -0.01 |
| rs16822383 | rs9490248 | rs9516391 | 11.01 | 0.0009 | 0.042 |  |  |  | -0.01 |
| rs13224531 | rs6990287 | rs2792752 | 10.91 | 0.001 | 0.067 |  |  |  | -0.01 |

Supplementary Table 6 Genes involved in functional enrichment analysis

| AADACL2-AS1 | AC093840.1 | ANXA13 | GPAM | NIM1K | RP11-63E5.6 |
| --- | --- | --- | --- | --- | --- |
| AC009117.1 | AC096992.1 | AP003398.2 | HCRTR2 | NPLOC4 | RP11-968A15.2 |
| AC009509.1 | AC104662.2 | APOOP2 | HIF1AN | NPSR1 | RPL12P4 |
| AC009869.1 | AC105252.1 | BX119904.1 | HMCN2 | NPSR1-AS1 | RUNX1 |
| AC010255.3 | AC105450.1 | C9orf84 | HSPD1P15 | PCP4 | RYR3 |
| AC022784.1 | AC106744.1 | CBX5 | LAMA2 | PHF21B | SAMSN1 |
| AC024909.1 | AC106800.1 | CDH13 | LCMT1 | PLAGL1 | SEPSECS-AS1 |
| AC025252.1 | AC117382.2 | CTC-210G5.1 | LINC00430 | PSG4 | SLC4A4 |
| AC026396.1 | AC123767.1 | CTC-441N14.4 | LINC00507 | PSG5 | SNX30 |
| AC026884.1 | ADAM10 | DGCR6 | LINC01376 | PSMC1P6 | SOX6 |
| AC034268.2 | ADAM18 | DNAJB8 | LINC01440 | RAPGEF4 | SPARC |
| AC068787.1 | AF127936.2 | DNER | LINC02199 | RF00017 | SPOCK1 |
| AC073592.2 | AL024498.2 | DPP10 | LINC-PINT | RF00019 | ST3GAL1 |
| AC073592.8 | AL137230.2 | EEFSEC | LYRM7 | RN7SKP190 | SYCP2L |
| AC079362.1 | AL138885.1 | FAM19A2 | MBD2 | RN7SKP93 | TAF15 |
| AC079380.1 | AL139317.5 | FAM230F | MCPH1 | RNU1-150P | TEMN3-AS1 |
| AC087379.1 | AL161716.1 | FAM76B | MELK | RNU6-1323P | TMPRSS15 |
| AC090515.3 | AL353595.1 | FAM83B | MGAT5 | RNU6ATAC21P | TPO |
| AC091895.1 | AL589740.1 | FAM87A | MIR4475 | RP11-166A12.1 | TPST1 |
| AC092100.1 | AL589826.1 | FARS2 | MMP28 | RP11-285A15.1 | TRIP12 |
| AC092364.1 | AL713851.1 | FBLN2 | MRPS35P3 | RP11-309P22.1 | UBBP5 |
| AC093277.1 | ALDH3B1 | FERMT2 | NDFIP1 | RP11-406O16.1 | UGGT1 |
| AC093462.1 | ANGPT2 | GNAL | NHSL1 | RP11-427M20.1 | VAV2 |
